# The palaeoenvironment of the Upper Cretaceous (Cenomanian–Turonian) portion of the Winton Formation, Queensland, Australia

**DOI:** 10.7717/peerj.5513

**Published:** 2018-09-07

**Authors:** Tamara L. Fletcher, Patrick T. Moss, Steven W. Salisbury

**Affiliations:** 1 Department of Ecosystem and Conservation Sciences, University of Montana, Missoula, MT, United States of America; 2 Department of Geography, University of Nevada—Reno, Reno, United States of America; 3 School of Earth and Environmental Sciences, University of Queensland, Brisbane, Australia; 4 School of Biological Sciences, University of Queensland, Brisbane, Australia

**Keywords:** Palaeoclimate, Reconstruction, Palaeoecology, Flora, Fauna, Diversity, Disturbance, Dinosaur, Mesozoic

## Abstract

The Winton Formation is increasingly recognised as an important source of information about the Cretaceous of Australia, and, more broadly, the palaeobiogeographic history of eastern Gondwana. With more precise dating and stratigraphic controls starting to provide temporal context to the geological and palaeontological understanding of this formation, it is timely to reassess the palaeoenvironment in which it was deposited. This new understanding helps to further differentiate the upper, most-studied portion of the formation (Cenomanian–Turonian) from the lower portions (Albian–Cenomanian), allowing a coherent picture of the ecosystem to emerge. Temperatures during the deposition of the Upper Cretaceous portion of the Winton Formation were warm, with high, seasonal rainfall, but not as extreme as the modern monsoon. The landscape was heterogeneous, a freshwater alluvial plain bestrode by low energy, meandering rivers, minor lakes and mires. Infrequent, scouring flood events were part of a multi-year cycle of drier and wetter years. The heavily vegetated flood plains supported abundant large herbivores. This was the final infilling of the great Eromanga Basin.

## Introduction

The Winton Formation of central-western Queensland provides a detailed insight into the continental biota of eastern Gondwana during the middle part of the Cretaceous Period, with deposition spanning from the upper Albian to the upper Cenomanian–lower Turonian (approximately 101–92 million years ago; Tucker et al., 2013; Tucker et al., 2016; Tucker et al., 2017). Its faunal assemblage includes evidence of a diverse range of invertebrates (insects, freshwater bivalves and gastropods), along with both aquatic and terrestrial vertebrates (dinosaurs, crocodyliforms, teleosts and dipnoan lungfishes, lizards, and turtles; Thulborn & Wade, 1979; Thulborn & Wade, 1984; Dettmann & Clifford, 1992; Hocknull, 1997; Kemp, 1997; Hocknull, 2000; Molnar & Salisbury, 2005; Hocknull & Cook, 2008; Scanlon & Hocknull, 2008; Dettmann, Clifford & Peters, 2009; Hocknull et al., 2009; Agnolin et al., 2010; Molnar, 2010; Romilio & Salisbury, 2011; Dettmann, Clifford & Peters, 2012; Romilio, Tucker & Salisbury, 2013; Berrell et al., 2014; Fletcher & Salisbury, 2014; Romilio & Salisbury, 2014; Poropat et al., 2015a; Poropat et al., 2015b; White et al., 2015a; White et al., 2015b; Poropat et al., 2016; White et al., 2016). At least 50 macrofossil plant taxa are present, from 10 orders, showing a co-dominance of araucariacean, podocarp and cupressacean conifers with early angiosperms (Bose, 1955; Whitehouse, 1955; White, 1966; Dettmann & Playford, 1969; Peters & Christophel, 1978; McLoughlin, Drinnan & Rozefelds, 1995; Chambers, Drinnan & McLoughlin, 1998; Martin, 1998; Pole, 1999; Pole & Douglas, 1999; Dettmann & Clifford, 2000; Pole, 2000a; Pole, 2000b; Clifford & Dettmann, 2005; Dettmann, Clifford & Peters, 2009; McLoughlin, Pott & Elliott, 2010; Dettmann, Clifford & Peters, 2012; Fletcher et al., 2014a).

Prior to 2013, the age of Winton Formation could only be constrained relatively, based mainly on palynological and biostratigraphic correlations (Cookson & Dettmann, 1959; Dettmann & Playford, 1969; Burger, 1970; Senior, Harrison & Mond, 1978; Frakes et al., 1987; Helby, Morgan & Partridge, 1987; Burger, 1990; Dettmann et al., 1992; Burger, 1993), and there was limited stratigraphic framework in which to place fossil-bearing localities. Work by Bryan et al. (2012), Tucker et al. (2013), Tucker et al. (2016), Tucker et al. (2017)) and Syme et al. (2016) has greatly improved the temporal framework of the Winton Formation through the determination of maximum ages for specific fossil-bearing localities using detrital zircon chronology along with more detailed stratigraphic, sedimentological and architectural analyses. It is now apparent that the Winton Formation was deposited over a span of at least 10–12 million years, during what can be regarded as the terminal phase of the Eromanga Sea, with regression from marine to continental ecosystems driving environmental change across the basin (Tucker et al., 2017). The tempo of this transition and how it affected the local flora and fauna has only recently begun to be appreciated and assessed (Syme et al., 2016; Tucker et al., 2017; Syme & Salisbury, 2018).

Given the pace at which new information on the age and stratigraphy of the Winton Formation has emerged in recent years, a reassessment of its changing palaeoenvironment using new data on its geology, hydrology, palaeoclimate, flora, and fauna, is timely. This is particularly the case for the Upper Cretaceous (Cenomanian–Turonian) portion of the Winton Formation, in which the bulk of the described fossil sites occur. A more refined model of the changing palaeoenvironmental conditions that existed during the time the Winton Formation was deposited will be of use not only for future research on the Winton Formation specifically but also for Cretaceous researchers more broadly. The refined models can also be used as a base from which future finds may be used to clarify, resolve or change our understanding of this increasingly important suite of ‘mid’ Cretaceous terrestrial localities, with the added advantage of considerably simplifying comparisons with other Cretaceous Gondwanan fossil assemblages.

## Survey Methodology

Multiple electronic searches were conducted from study conception to February 2018, with no language or period restrictions, in online databases including the ISI/Web of Science, Scopus and Google Scholar. Initial search terms included ‘Winton Formation’, as well as combinations of ‘Cretaceous’, ‘Queensland’, and ‘Australia’. The search included materials in peer-reviewed journal articles, geological reports, conference proceedings and academic books. Non-peer-reviewed sources such as unpublished theses, conference abstract volumes and popular articles were not included. We also searched the reference section of these publications, which aided discovery of older sources, particularly where the age and name of units has been reassigned since publication. Materials were included that were considered to inform the authors on the palaeoenvironment. Articles related to the Winton Formation, but on derived topics (e.g., biomechanics of Winton Formation dinosaurs), were only included if they also contributed to the broader palaeoenvironmental reconstruction.

## Geology

**Figure 1:**
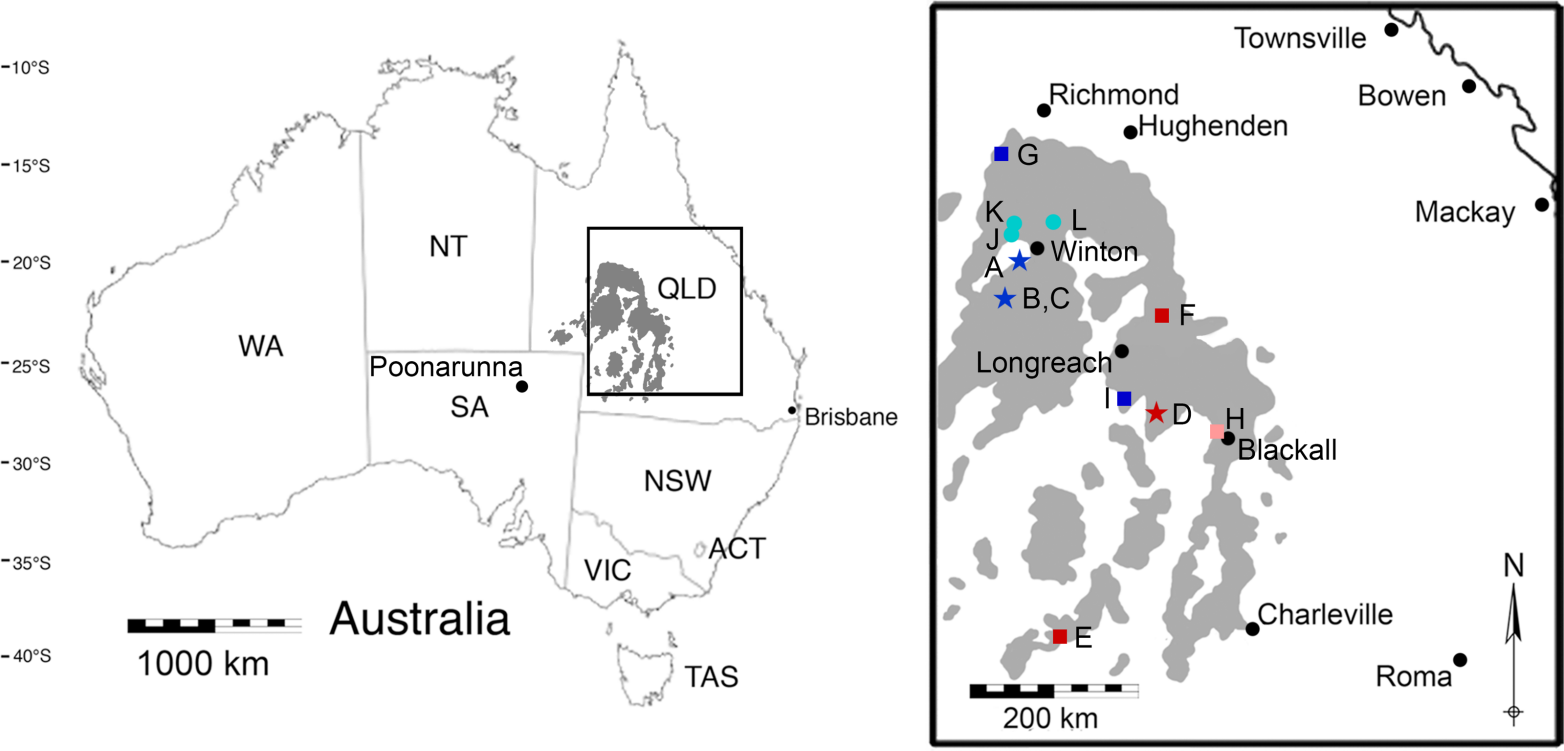
A map of the outcropping of the Winton Formation in Queensland, Australia. This map of Australia shows the outcropping of the Winton Formation in Queensland (in grey), focusing on the fossiliferous area around Winton (enlarged; adapted from Fletcher et al., 2014a). Coloured symbols indicate sites dated by detrital zircons (dark blue/red) or sites stratigraphically correlated to sites dated by detrital zircons (light blue/pink) in Tucker et al. (2013) and Syme et al. (2016). Stars indicate surface samples; squares indicate Geologic Survey of Queensland (QSQ) cores; circles are correlated surface exposures. (A) Bladensburg National Park; (B) Lark Quarry Conservation Area—Eureaka sample; (C) Lark Quarry Conservation Area—Hades Hill sample; (D) Isisford concretion; (E) GSQ Eromanga 1; (F) GSQ Longreach 1-1B; (G) GSQ McKinlay 1 (Sample 2 and 16); (H) GSQ Blackall 1; (I) GSQ Blackall 2; (J) Lovelle Downs/Elderslie Station (QM L311); (K) Alni Station; (L) Belmont Station. QLD, Queensland; NSW, New South Wales; ACT, Australian Capital Territory; VIC, Victoria; SA, South Australia; NT, Northern Territory; QM L, Queensland Museum Locality.

**Figure 2:**
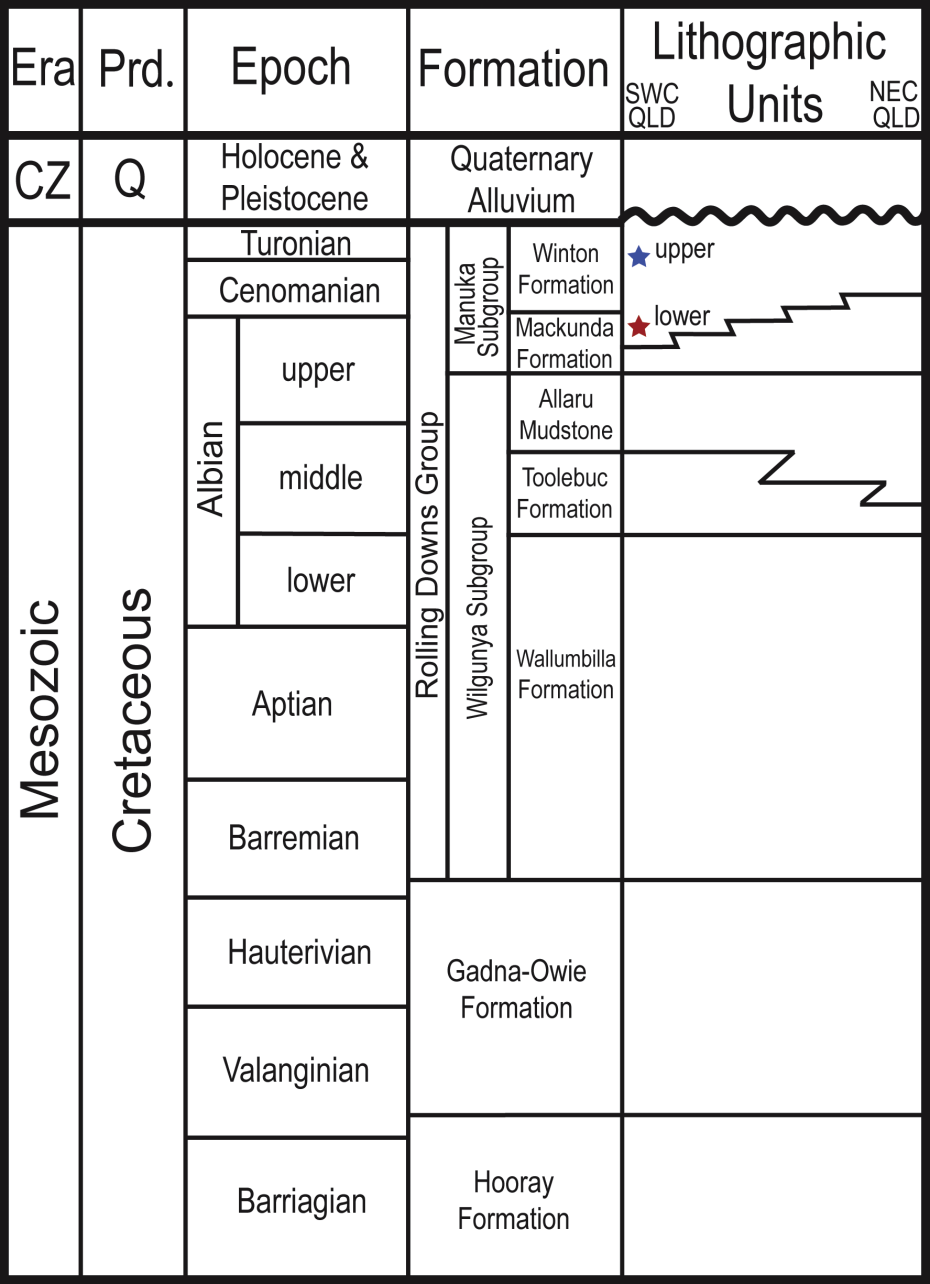
Stratigraphic column showing the relative position of the Upper and Lower Cretaceous portions of the Winton Formation.

The Winton Formation covers a large geographic area of western Queensland, northeast South Australia and northwest New South Wales (Fig. 1; Gray, McKillop & MeKellar, 2002) and has been the subject of interest for both economic geology and basic research (Table 1). At the time the Upper Cretaceous portion of the formation was deposited, central western Queensland was at ∼50°S palaeolatitude (Li & Powell, 2001). The Winton Formation conformably overlies the marine Mackunda Formation, forming the uppermost unit of the Manuka subgroup within the Rolling Downs Group in the intracratonic Eromanga Basin, and its surface is either exposed or overlain locally by Paleogene or younger strata (Fig. 2; McLoughlin, Pott & Elliott, 2010). Deep weathering of the Winton Formation has oxidised palynomorphs in those parts close to the surface, limiting the capacity to estimate the youngest age of deposition (McLoughlin, Pott & Elliott, 2010). The formation, as a whole, consists of volcanolithic sandstones, likely derived from the Whitsundays Volcanic Provence (Bryan et al., 1997; Bryan et al., 2000; Bryan et al., 2012; Tucker et al., 2013; Tucker et al., 2016; Tucker et al., 2017), and facies that are complex and repetitive including fine- to medium-grained feldspatholithic or lithofeldspathic arenite, siltstone, mudstone, and claystone (Fielding, 1992; Romilio & Salisbury, 2011; Romilio, Tucker & Salisbury, 2013; Tucker et al., 2013; Syme et al., 2016; Tucker et al., 2016; Tucker et al., 2017) with very minor coal seams (Senior, Harrison & Mond, 1978).

**Table 1:** Key literature on the geology of the Winton Formation.

Topic		Reference
Sedimentology		Dunstan (1916)
		Wopfner (1963)
		Vine et al. (1967)
		Casey (1970)
		Senior & Mabbutt (1979)
		Coote (1987)
		Fielding (1992)
		Draper (2002)
		Tucker et al. (2017)
Stratigraphy		Dunstan (1916)
		Exon & Senior (1976)
		Senior, Harrison & Mond (1978)
		Henderson & Stephenson (1980)
		Fielding (1992)
		Draper (2002)
		Gray, McKillop & MeKellar (2002)
		Mavromatidis & Hillis (2005)
		Tucker et al. (2017)
Age	Palynology	Dettmann & Playford (1969)
		Burger (1986)
		Frakes et al. (1987)
		Helby, Morgan & Partridge (1987)
		Burger (1990)
		Burger (1993)
		Partridge (2006)
		Clifford & Dettmann (2005)
		Dettmann, Clifford & Peters (2009)
		Dettmann, Clifford & Peters (2012)
	Geo-chronological methods	Bryan et al. (2012)
		Tucker et al. (2013)
		Tucker et al. (2016)
		Tucker et al. (2017)
Source material		Bryan et al. (1997)
		Bryan et al. (2000)
		Bryan et al. (2012)
		MacDonald et al. (2013)
		Tucker et al. (2013)
		Tucker et al. (2016)
		Tucker et al. (2017)
Geomorphology and Tectonics		Casey (1967a)
		Casey (1967b)
		Casey (1970)
		Hawkins & Harrison (1978)
		Mathur (1983)
		Moss & Wake-Dyster (1983)
		Moore & Pitt (1984)
		Wopfner (1985)
		Finlayson, Leven & Etheridge (1988)
		Murray, Scheibner & Walker (1989)
		Green et al. (1991)
		Cartwright & Lonergan (1997)
		Veevers (2000)
		Veevers (2006)
		Hower (2011)
		MacDonald et al. (2013)
		Tucker et al. (2013)
		Moya et al. (2015)
		Tucker et al. (2016)

On the basis of detrital zircon chronology, stratigraphy, sedimentology, and architectural analysis, the Winton Formation is now informally divided into Upper and Lower Cretaceous parts (Tucker et al., 2013; Tucker et al., 2016; Tucker et al., 2017). Fossil-bearing localities in the greater Winton area, such as those on Elderslie, Belmont and Lovelle Downs Stations, Lark Quarry Conservation Area, and Bladensburg National Park, come from a portion of the Winton Formation that was deposited during the early Late Cretaceous (late Cenomanian–early Turonian, ∼93.9 Ma), with a number of distinct horizons and facies associations that are fairly continuous across the region (Tucker et al., 2013; Tucker et al., 2017). Other Winton Formation fossil-bearing localities, such those in the Isisford area (Salisbury et al., 2006; Berrell et al., 2014; Syme et al., 2016; Syme & Salisbury, 2018) were deposited some 10 million years earlier during late Early Cretaceous (late Albian to early Cenomanian, ∼103–100.5 Ma). Using the same approach, Tucker et al. (2016) were able to constrain the upper Mackunda Formation to the latest Albian (∼104–102 Ma). The onset of deposition of the lowest parts of the Winton Formation can therefore be no older than this. Fossil-bearing sites in the Lower Cretaceous portion of the Winton Formation, near Isisford, are stratigraphically much lower than those that occur in the Upper Cretaceous portion despite having similar modern altitudes to localities farther north (Winton area) and south (Eromanga) due to local faulting (Tucker et al., 2013; Tucker et al., 2017). Whereas the younger sites occur in sediments indicative of a freshwater alluvial plain (Exon & Senior, 1976; Senior, Harrison & Mond, 1978; Moore & Pitt, 1984; Fielding, 1992; Romilio, Tucker & Salisbury, 2013; Tucker et al., 2017), the older ones are thought to have been deposited in brackish waters in a tidally influenced fluvial-deltaic setting near the shores of the still present Eromanga Sea (Berrell et al., 2014; Syme et al., 2016; Tucker et al., 2017). Accordingly, Tucker et al. (2017) informally refer to this part of the Winton Formation as the ‘lower Winton Formation’. Earlier studies of core samples (e.g., Pole, 1999; Pole, 2000a; Pole, 2000b) did not have as well constrained dates, but demonstrated that there is up to 300 m of stratigraphic thickness of fossil-bearing Winton Formation deposition below the now dated 93 million year old sites, corroborating the ‘upper’ designation for this part of the formation proposed by Tucker et al. (2017). For example, the cores from GSQ Maneroo, although shallower, are stratigraphically equivalent to deeper samples from GSQ Thargomindah (Pole, 1999; Pole & Douglas, 1999; Pole, 2000a; Pole, 2000b) as a result of uplift of the Maneroo Platform. Based on palynology, these units were likely deposited at close to the Albian-Cenomanian boundary, and appear to contain a different plant community that is overwhelmingly dominated by conifers (Pole, 2000a) distinct from the conifer-angiosperm co-dominant outcropping localities of the Upper Cretaceous portion of the Winton Formation (McLoughlin, Pott & Elliott, 2010).

## Hydrology

It is clear from surface sediment localities in the greater Winton region that the Upper Cretaceous portion of the Winton Formation was derived from terrestrial and freshwater depositional environments (Table 2; Tucker et al., 2013; Syme et al., 2016; Tucker et al., 2017). This is also confirmed by the presence of freshwater gastropods (Cook, 2005) and freshwater bivalves (Hocknull, 1997; Hocknull, 2000). Although some earlier studies (e.g., Thulborn & Wade, 1979; Thulborn & Wade, 1984) proposed that lakes may have been present at sites such as near Lovelle Downs Station (QM L311) and the Lark Quarry Conservation Park, more recent work has shown lacustrine sedimentation did not occur either before and during the formation of the track-bearing surface at Lark Quarry (Romilio, Tucker & Salisbury, 2013) and that lakes were a relatively minor component of a landscape that primarily comprised channels and floodplains (Tucker et al., 2013; Tucker et al., 2017). The great thickness of the Winton Formation (up to 1,000 m in some areas; see Tucker et al., 2013; Tucker et al., 2017), suggests a massive river system that, over at least 10 million years and in various forms, filled the epicontinental Eromanga Sea, whilst during distinct periods, also flowing south-west as part of the continental Ceduna River system (Lloyd et al., 2016). The sediments directly associated with the plant-bearing horizons that are considered in this study were predominantly siltstone (McLoughlin, Drinnan & Rozefelds, 1995), with mudstone and dolostone also present at QM L311 (Dettmann, Clifford & Peters, 2009). This, added to the sedimentary features identified in outcrops, including current ripple cross-lamination, trough and tabular cross-stratification (Tucker et al., 2013; Tucker et al., 2017), and the probable ox-bow lakes (Hocknull et al., 2009; Tucker et al., 2017) suggests a meandering river system, and has been confirmed by the more detailed sedimentological assessments presented in Tucker et al. (2017).

**Table 2:** Key literature that informs the interpretation of the hydrology of the Upper Cretaceous portion of the Winton Formation.

Evidence type	Feature	Water chemistry	Flow rate	Winton citation
Sedimentology	Sedimentary structures such as crossbedding, ripple marks etc		Expansive fluvial floodplain system, changing over time with waxing and waning flow to low flow.	Tucker et al. (2013), Tucker et al. (2017)
	Lithology such as fine-grained sandstones, siltstones and mudstones		Low flow river with minor lacustrine component including oxbow lakes	Tucker et al. (2013)
	Lithology and sedimentary structures		Main channel deposits with flood plains and small lakes suggesting variability temporally and geographically	Fielding (1992)
Faunal	Gastropods *Melanoides sp.*	Freshwater but some species saline tolerant	Widely tolerant	Cook (2005)
	Bivalves *Alathyria jaqueti* *Megalovirgus wintonensis* *Hyridella macmichaeli*	Freshwater	Widely tolerant	Hocknull (1997), Hocknull (2000)
	Lungfish *Metaceratodus ellioti* *Metaceratodus wollastoni*	Freshwater	Still to slow flow	Kemp (1997)
	Turtles	Freshwater	Widely tolerant	Molnar (1991)
	Crocodyliforms Crocodyliformes indet.	Fresh or saline	Widely tolerant	Hocknull et al. (2009)

The movement, distribution, and quality of water and how these characters change through time, are important determinants of the biota and ecological processes in fluvial systems (e.g., Gorman & Karr, 1978; Allan, 1995; Richter et al., 1996; Matthaei, Peacock & Townsend, 1999). Thus, the composition of biota left by a system can inform interpretation of the hydrology of the system. The presence or absence of animals that have specific hydrological requirements are the most useful faunal evidence. The most informative amongst the fauna is the presence of lungfish tooth plates (Kemp, 1997). The Australian lungfish *Neoceratodus forsteri* Krefft, 1870, a living example from the family Neoceratodontidae to which the Cretaceous lungfish found in the Winton Formation belong, live in low-flow to still waters and require a low-flow regime in spring for egg laying on shallow-water plants (Espinoza, Marshall & McDougall, 2013). The African lungfishes, of the genus *Protopterus* Owen, 1839*,* also live in low energy environments such as swamps, marshes and lakes, with a preference for shallow, inshore waters, with only some large adults found offshore in large lake systems (Chapman et al., 1996; Goudswaard, Witte & Chapman, 2002). The final genus of lungfish with extant examples, *Lepidosiren* from South America, also live in swamps and lakes (Johansen & Lenfant, 1967), cumulatively suggesting a conserved habitat preference. No evidence of lungfish has been found among the semi-articulated teleosts (Berrell et al., 2014) of the marginal marine-deltaic system that formed the Lower Cretaceous (upper Albian–lower Cenomanian) portion of the Winton Formation (Tucker et al., 2017), despite substantial sampling. This suggests that modern habitat preferences hold for Cretaceous lungfish (Berrell et al., 2014). Other key aquatic fauna from the Upper Cretaceous portion of the Winton Formation have wide tolerances amongst their modern analogues and so were not informative. For example, modern crocodiles such as *Crocodylus johnstoni* Krefft, 1873—an analogue for *Isisfordia duncani*—live in a wide range of hydrological environments (Webb, Manolis & Sack, 1983) including tidal and saline waters (Messel et al., 1981). The brackish conditions of the depositional setting associated with the Lower Cretaceous portion of the Winton formation that was inhabited by *I. ducani* (Syme & Salisbury, 2018) is consistent with this range of environmental tolerance. Freshwater turtles have differing responses to flow rates due to how they use the habitat, with some preferring higher velocity water flow and others slow (Lenhart, Naber & Nieber, 2013). A slow, but flowing freshwater hydrologic regime, such as a meandering river system, is in keeping with the geology of the Upper Cretaceous sediments, the composition of the aquatic fauna, and the gradual dip of the infilling flood plain.

## Palaeoclimate

Previous palaeoclimatic assessments of the Winton Formation treated it as one palaeoenvironment, generally regarded as warm temperate (e.g., Dettmann & Playford, 1969), and although the presence of coal suggested humidity, Dettmann et al. (1992) interpreted the composition of the floral community to be indicative of overall dry conditions. The refined higher resolution dating of the floral and faunal communities and the addition of fossils discovered in the last 25 years, provides an opportunity to investigate the climate at a higher resolution.

Recently, quantitative methods have been applied to investigate the palaeoclimate of the Upper Cretaceous portion of the Winton Formation in greater detail (Table 3). These studies have shown that the palaeoclimate of these localities was generally warm (∼16 °C mean annual temperature) and wet (>1,300 mm mean annual precipitation) as estimated by the foliar physiognomic methods of Leaf Area Analysis, Leaf Margin Analysis and Climate Leaf Analysis Multivariate Program, and mutual climatic range methods from the floral assemblage (Fletcher, Moss & Salisbury, 2013; Fletcher et al., 2014b). Although precipitation appears to have been high and seasonal, it was not as high as what is experienced in some high elevation modern forests today, nor with extremes of wet and dry as is associated with the modern monsoon. For example, in modern Darwin, which is subject to monsoonal weather systems, the cumulative three wettest months receive 1,120 mm on average and the three driest receive 8.1 mm (BOM, http://www.bom.gov.au/climate/data-services/, date accessed: 26/01/2014). Compare this with the Upper Cretaceous portion of the Winton Formation, which is estimated to have received 630 ± 206 mm during the three wettest months and 268 ± 137 mm during the three driest months (Fletcher et al., 2014b). The growth indices of the fossil wood also indicate that climate variables differed between years to the extent that it had a notable influence on ring width in the podocarp-like wood at the locality (Fletcher, Moss & Salisbury, 2015). This variability appears to be non-random and oscillatory, and may indicate that phenomena similar to the Pacific Decadal Oscillation (Mantua & Hare, 2002; McGowan et al., 2009) were driving patterns of climate in the early Late Cretaceous.

**Table 3:** Key literature that informs the palaeoclimatic interpretation of the Upper Cretaceous portion of the Winton Formation.

Climate variable		Estimate	Evidence source	Reference
Temperature	Mean annual	>16 °C	Crocodilians	Hocknull et al. (2009)
		>16 °C (tropical to subtropical)	Lungfish	Kemp (1997)
		16.4 °C ± 5 °C	Leaf Margin Analysis - LQ	Fletcher, Moss & Salisbury (2013)
		16 °C ± 2 °C	CLAMP - LQ	Fletcher, Moss & Salisbury (2013)
		13.6 ± 2 °C	CLAMP - QM	Fletcher et al. (2014b)
		15.9 ± 1.2 °C	Bioclimatic Analysis	Fletcher et al. (2014b)
	Warmest mean monthly	20.3 ± 2 °C	Bioclimatic Analysis	Fletcher et al. (2014b)
		21.2 ± 2.7 °C	CLAMP - QM	Fletcher et al. (2014b)
		>17.5 °C	Freshwater turtles	Molnar (1991)
	Coldest mean monthly	10.3 ± 1 °C	Bioclimatic Analysis	Fletcher et al. (2014b)
		6.7 ± 3.4 °C	CLAMP - QM	Fletcher et al. (2014b)
Growth Season Length		9 months	CLAMP - LQ	Fletcher, Moss & Salisbury (2013)
		7.8 ± 1.1 months	CLAMP - QM	Fletcher et al. (2014b)
Precipitation	Mean annual	1,321 mm + 413 mm–315 mm	Leaf Area Analysis –LQ	Fletcher, Moss & Salisbury (2013)
		1,646 ± 370 mm	Bioclimatic Analysis	Fletcher et al. (2014b)
	Growth season	1,073 ± 483 mm	CLAMP - LQ	Fletcher, Moss & Salisbury (2013)
	Growth season	1,289 ± 483 mm	CLAMP - QM	Fletcher et al. (2014b)
	3 driest months	268 ± 137 mm	CLAMP - QM	Fletcher et al. (2014b)
	3 wettest months	630 ± 206 mm	CLAMP - QM	Fletcher et al. (2014b)
Inter and intra-annual variability		Wet season double dry season precipitation	CLAMP	Fletcher et al. (2014b)
		‘High seasonality’	Ring markedness index	Fletcher, Moss & Salisbury (2015)
		High inter-annual variability	Mean sensitivity >0.3 (ring index)	Fletcher, Moss & Salisbury (2015)
Sea Surface temperatures		‘Warm’	Dolichosaurs	Scanlon & Hocknull (2008)

In addition to botanical indicators, elements of the fauna are climatically informative. Crocodyliform remains have been reported in the Upper Cretaceous portion of the Winton Formation at AOD L-85, on Elderslie Station approximately 60 km north-wet of Winton (Hocknull et al., 2009). Assuming this material is assignable to *Isisfordia duncani* or a closely related species, and if eusuchians are accepted as paleothermometers (Markwick, 1994; Markwick, 1998) their presence supports a MAT of ≥16 °C. Modern lungfish, as analogues for *Metaceratodus ellioti* (Kemp, 1997) from the Upper Cretaceous portion of the Winton Formation, have a distribution limited to the tropics and subtropics. Freshwater turtles from the upper portion of the Winton Formation (Molnar, 1991) also suggest warmth as even cold-adapted modern turtles require warmest mean monthly temperatures over 17.5 °C (Tarduno et al., 1998). Finally, the presence of dolichosaurs in the Upper Cretaceous portion of the Winton Formation suggests sea surface temperatures conducive to oceanic dispersal, in keeping with warming through to the end of the Turonian (Scanlon & Hocknull, 2008).

## Flora

### Macrofloral composition

**Figure 3:**
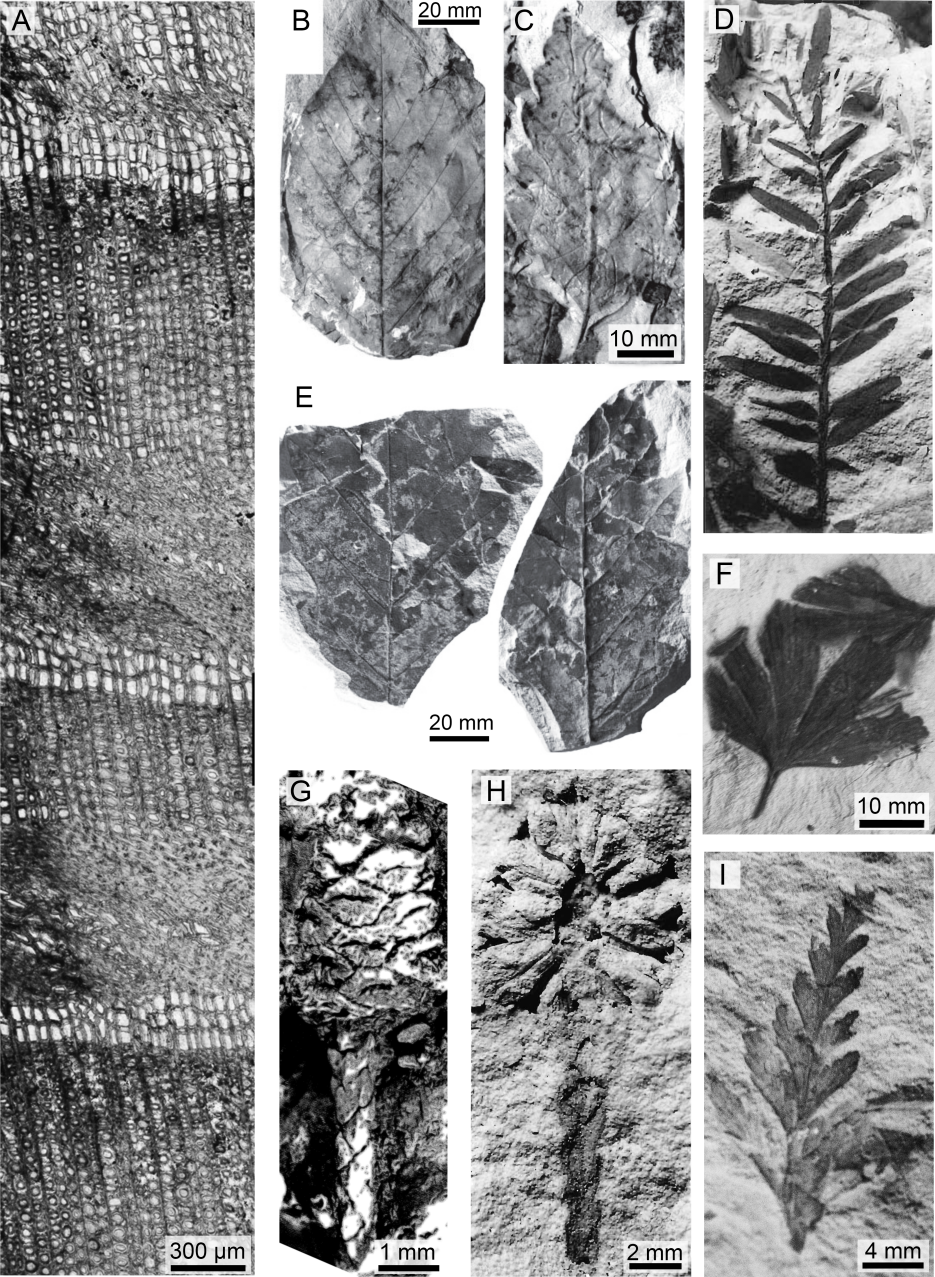
A sample of the variety of taxa, organs, and preservation of plant fossils from the Upper Cretaceous portion of the Winton Formation. (A) A transverse section of *Protophyllocladoxylon owensii* Fletcher et al. showing repeated crushing in early wood, QM F44336 (Fig. 2A; Fletcher, Moss & Salisbury, 2015)^1^; (B) Winton Formation Leaf morphotype B, QM F32459 (Fig. 3D; Fletcher, Moss & Salisbury, 2013); (C) Winton Formation Leaf morphotype C, QM F32510 (Fig. 2D; Fletcher, Moss & Salisbury, 2013); (D) *Araucaria sp*. cf. *A. mesozoica* Walkom foliage, QM F32561 (Fig. 8F; McLoughlin, Drinnan & Rozefelds, 1995); (E) Part and counterpart of leaf impression from Lark Quarry Conservation Area, UQL-LQ-III 24 (Fig 2A; Fletcher, Moss & Salisbury, 2013); (F) *Ginko wintonensis* McLoughlin et al. holotype leaf impression, QM F32478b (Photo: T.F.); (G) *Austrosequoia wintonensis* Peters and Christophel, silicified ovulate cone and terminal leaves, QM F9509 (Fig 2; Peters & Christophel, 1978); (H) cf. *A. wintonensis* longitudinal imprint of a terminal cone and stem with scale-like leaves, QM F3250 (Fig. 11G; McLoughlin, Drinnan & Rozefelds, 1995); (I) *Sphenopteris sp.* fragmentary frond, UQL-LQ-III 2 (Photo: T.F.).1 Reprinted from Palaeogeography, Palaeoclimatology, Palaeoecology, 417, T. Fletcher, P.T. Moss, S.W. Salisbury, Wood growth indices as climate indicators from the Upper Cretaceous (Cenomanian–Turonian) portion of the Winton Formation, Australia, 35–42., Copyright 2015, with permission from Elsevier.

Although the geological evidence suggests a broadly connected and coeval sedimentary layer, some of the plant-bearing fossil localities in the Upper Cretaceous portion of the Winton Formation are over 100 km apart and are not likely to represent one continuous flora. Although regarding the Winton Formation as a whole, McLoughlin, Pott & Elliott (2010) noted the variation in plant assemblages across sites in the 16 major studies of the Winton macroflora. Indeed, the Lark Quarry locality conifer leaf impressions suggest a dominance of multiple species of araucarians (Fletcher, Moss & Salisbury, 2013), while at QM L311 *Protophyllocladoxylon owensii* Fletcher et al., with likely podocarp affinities, appears dominant, both in terms of wood remains and from a frequency count of pollen (Dettmann, Clifford & Peters, 2009; Fletcher, Moss & Salisbury, 2015). This suggests a heterogeneous distribution of plant communities across the landscape (Fig. 3).

The podocarp-dominated plant community at QM L311 is of particular palaeoecological interest as such communities are rare in modern systems. In modern forests podocarps are universally slow growing and are usually only a significant component in communities growing on nutrient-poor soils (Coomes & Bellingham, 2011). These species mitigate the problem of low nutrient availability by limiting mineral loss through long leaf retention times (Monk, 1966) and through nutrient uptake via endomycorrhizal symbionts (Baylis, McNabb & Morrison, 1963; Baylis, 1969; Russell, Bidartondo & Butterfield, 2002). Indeed, Baylis, McNabb & Morrison (1963) found that on poor nutrient soils, podocarps are dependent on mycorrhizal fungi. Cantrill & Falcon-Lang (2001) reported impressions of nodules on roots similar to those seen in podocarps as early as the Albian. Thus, it is likely that associations between conifer roots and fungi were present in the Late Cretaceous. In addition, it was found that *P. owensii* had a likely leaf retention time (∼3–6 years) similar to modern podocarps, including those that grow on low nutrient soils (Fletcher, Moss & Salisbury, 2015). However, the fluvial plains of volcanically derived sediments that constitute the upper portion of the Winton Formation were unlikely to be low in nutrients, and thus modern centres of podocarp diversity, such as New Caledonia (Contreras-Medina & Vega, 2002; Mutke & Barthlott, 2005) are unlikely to be good analogues.

The exceptions to this low-nutrient rule are the three podocarp species that dominate the forests of the alluvial flood plains in south-western New Zealand: *Dacrycarpus dacrydioides* (A.Rich.) de Laub., *Prumnopitys taxifolia* (Sol. ex D.Don) de Laub., and *Podocarpus totara* G.Benn. ex D.Don (Coomes & Bellingham, 2011). The success of these species can be linked to catastrophic disturbances (earthquakes and floods) by their single age stands (Duncan, 1991; Duncan, 1993; Wells, Duncan & Stewart, 2001) and by the effect of long periods without disturbance. Where Wardle (1974) was able to locate undisturbed forest, canopy spaces generated by windfall etc. of the dominant *D. dacrydioides* were replaced by tree ferns and broad-leafed angiosperm species. Otherwise, a frequency of disturbance in the order of hundreds of years is sufficient for these podocarps to remain dominant due to their longevity (Coomes & Bellingham, 2011). Holloway (1954) described these resulting podocarp forests as patchworks containing many kinds of podocarp communities, some of which were specialised to particular sites and soils, as well as different severity of disturbance (Duncan, 1993).

Given that this portion of the Winton Formation comprises alluvial sediments that are generally considered nutrient rich, it seems likely the podocarp-dominated forests were driven by successional patterns as part of a broader disturbance regime. Thus, despite the differences in climate, the south-western New Zealand podocarp forests may be the best modern analogue for the Upper Cretaceous podocarp-dominated flora. That no modern flora is a good analogue for the Winton flora may not indicate taphonomic bias, but rather that the role of podocarps in the depositional setting of the Upper Cretaceous portion of the Winton Formation is taken by tall woody angiosperms in modern systems.

A Cretaceous example of a similarly heterogeneous community structure, with a dominant podocarp component, is described by Falcon-Lang, Cantrill & Nichols (2001). Within the Lower Cretaceous (upper Albian) Triton Point Member of the Neptune Glacier Formation on Alexander Island, there appears to be two distinct communities based around a braided alluvial plain system and a meander-belt coastal plain, yet both comprise a number of sub-communities: broken podocarp woodland, gymnosperm-fern-angiosperm thickets, cycadophyte-conifer scrub, and liverwort mats in the braid-plain system, and podocarp-araucarian climax forest, fern thickets/conifer woodland, and ginkgo-conifer scrub in the coastal meander-belt system (Falcon-Lang, Cantrill & Nichols, 2001). Although the composition of the plant communities and the kind of fluvial setting in the Upper Cretaceous portion of the Winton Formation is somewhat different from these, it provides a framework to understand the difference in floral composition between sites. The general low diversity may suggest we do not have climax forest preserved amongst the Upper Cretaceous plant localities, although such sites may have existed within the palaeo-landscape. QM L311 may represent an ecosystem similar to the broken podocarp woodland of Falcon-Lang, Cantrill & Nichols (2001), or the podocarp dominated alluvial plains and wetlands of south-western New Zealand (Wardle, 1974; Duncan, 1991; Duncan, 1993; Wells, Duncan & Stewart, 2001).

Compared with the majority of sites in the upper portion of the Winton Formation, QM L311 is also unusual in its preservation and lithology. The majority of sites of this age are now thought to have been deposited in meandering river systems and flood plains, comprising predominantly leaf impressions in siltstone. In contrast, the cones, fruits and palynomorphs of QM L311 are silica-replaced and preserved in silica-rich claystone, suggesting a very slow flow or lacustrine environment. Although the taphonomic history of these localities has not been systematically evaluated, it appears the material was deposited in a silica-rich, anaerobic environment (Dettmann, Clifford & Peters, 2009). Given there is no evidence for volcanic or hydrothermal conditions that are thought to cause rapid silicification (Spicer, 1991; Locatelli, 2017), it is likely that anaerobic conditions were stable such that decay was slow enough to allow slow silicification. This suggests a lacustrine deposition and requires that either the lake did not overturn and was deep compared to its surface area (Hutchinson, 1957) or that it had a high perimeter to surface area, resulting in high organic input and limited surface gas exchange (Spicer, 1991). Given that the floral material entering a lake generally reflects the waterside flora (Greenwood, 1991), QM L311 likely reflects a local snapshot of the vegetation. Some podocarps survive well in swampy, poorly drained soils that may have occurred around the lake edge, and so may dominate the local signal.

The somewhat more diverse leaf impression assemblages of the Lark Quarry plant localities and of the localities from which McLoughlin, Drinnan & Rozefelds (1995) and McLoughlin, Pott & Elliott (2010) sourced material, may reflect more diverse, perhaps more mature, stands in which succession has taken place, more intensely sampled material, the lumping of these sites of similar deposition environments, or the increased natural sampling of a flora that occurs in a stream system as opposed to a lake (Burnham, 1989; Greenwood, 1991). Fluvial systems are biased to the river edge flora (Christophel & Greenwood, 1988) and thus the higher representation of angiosperm leaves may derive from their dominance of that niche. Early angiosperms are considered weedy in their growth habit (Royer et al., 2010) and common to disturbed, moist sites such as stream edges (Feild et al., 2004; Feild & Arens, 2005). Although most of the angiosperm leaf impressions cannot be precisely identified, many are assigned to Fagales, with the most common reminiscent of Cretaceous *Nothofagus* from New Zealand, and another to a Cretaceous leaf type from New Zealand tentatively assigned to Betulaceae (McLoughlin, Drinnan & Rozefelds, 1995). According to recent molecular studies (Xiang et al., 2014), the upper portion of the Winton Formation was deposited at approximately the time of the first diversification of Fagales, but prior to the divergence of Nothofagaceae and Betulaceae proper. The broad distribution of the Fagales, uncertainty in assignation and timing of diversification generally proclude further ecological interpretation based on these identifications, as opposed to those based on the leaf traits for early angiosperms more broadly. Similarly, the flower *Lovellea wintonensis* from QM L311, is assigned to Laurales, a diverse group with representatives from the tropics to the temperate regions. The higher representation of araucarians over podocarps in that setting may indicate very long leaf retention times in the podocarp species thus biasing against their proportional preservation, that these localities had not been subject to disturbance, for example by flood, as recently as QM L311 and thus succession taken place, or that the fluvial flora’s soils were better drained than those near QM L311. Either way, the discrepancy in floras is likely a true signal, in that it represents a heterogeneous landscape comprising various plant sub-communities, likely determined by abiotic factors such as disturbance, depositional environment and local topography.

### Diversity

**Figure 4:**
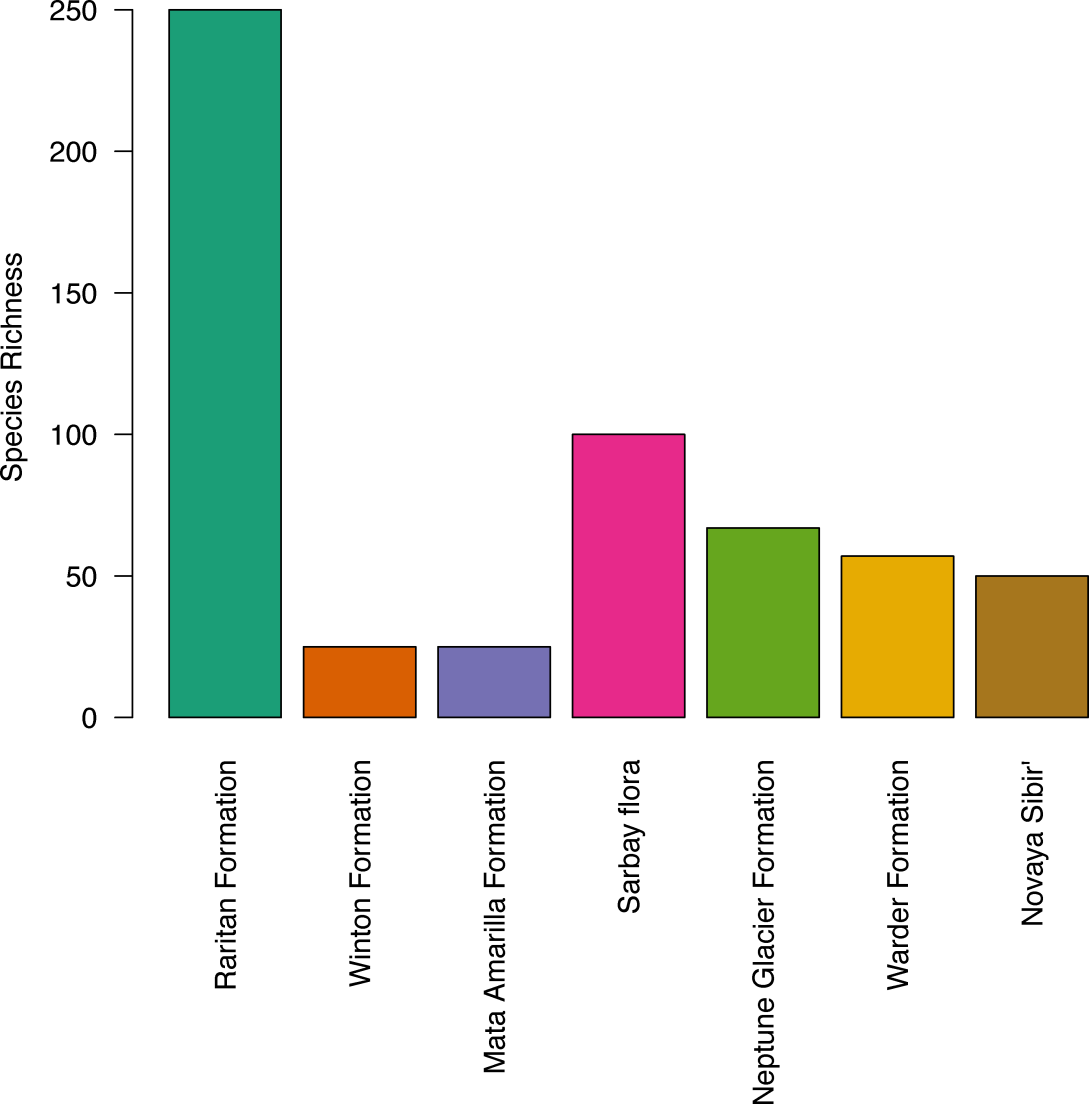
Histogram of species richness at fossil sites referred to in the text, from low to high latitude.

The apparent diversity of the macroflora of the Upper Cretaceous portion of the Winton Formation (<30 species; Table 4), is low when compared with many similar latitude and high latitude broadly coeval floras of both the Northern (Upper Cretaceous (Turonian) Raritan Formation, USA – 250+ species (Knowlton, 1919; Martínez-Millán, Crepet & Nixon, 2009 and references within); Upper Cretaceous (Cenomanian–Turonian) Sarbay flora, Kazakhstan – 100+ taxa (Frumin & Friis, 1999; Frumin, Eklund & Friis, 2004); Upper Cretaceous (Turonaian) Novaya Sibir’, Russian High Arctic – ∼50 species (Herman & Spicer, 2010)) and Southern Hemisphere (‘mid’-Cretaceous (Late Albian–Cenomanian) Warder Formation, New Zealand – 57+ species (Parrish et al., 1998); Lower Cretaceous (upper Albian) Triton Point member of the Neptune Glacier Formation, Antarctica – 67 species (Falcon-Lang, Cantrill & Nichols, 2001)) especially if accounting for the latitudinal biodiversity gradient (Fig. 4). The most similar comparison found is that of the Upper Cretaceous portion of the Winton Formation flora to the Upper Cretaceous (Cenomanian–Coniacian) upper Mata Amarilla portion of Mata Amarilla Formation, Argentina. The two flora compare well in sedimentary environment—an inland flood-plain—and richness of angiosperm morphotypes—10 morphotypes—though Iglesias et al. (2007), note the large difference in the proportion of plant groups preserved (gymnosperms more frequent in the Winton flora) and differences in the size, shape, and diversity of forms of the angiosperm leaves.

#### Causes of low diversity

A potential cause of the low diversity is sampling intensity. Fletcher, Moss & Salisbury (2013) investigated specimens from two new sites at Lark Quarry Conservation Area (Fig. 1; Romilio & Salisbury, 2011) and found no new taxa when compared with the known <30 species from 12 sites in the Upper Cretaceous portion of the Winton Formation (Table 4). Ideally, rarefaction would be conducted to assess the impact of sampling; however, not all of the information necessary for comparative analysis is available. In a simplified system, if additional, as-yet unidentified species were present in the total sample pool at Lark Quarry, to have a significant probability (≥5%) of finding none of them in 150 samples, the new species would need to comprise less than 2% of the total sample pool (0.98^150^ = 0.048). By comparison, Iglesias et al.’s (2007) revision of the flora from the Cenomanian–Coniacian Mata Amarilla Formation report on >500 leaf specimens, >300 from the Mata Amarilla Level. While the use of macrofossils in palaeoecological studies of diversity is an area in need of greater research (Birks et al., 2016), the probability suggests it is reasonable that other factors may have contributed to the apparent low diversity of the flora in the Upper Cretaceous portion of the Winton Formation.

**Table 4:** Described elements of the macroflora of the Upper Cretaceous portion of the Winton Formation.

Fossil type		Taxonomic group	Name	Reference
Wood		Pinophyta; Podocarpaceae	*Protophyllocladoxylon owensii*	Fletcher et al. (2014a)
Flower		Magnoliophyta; Laurales	*Lovellea wintonensis*	Dettmann, Clifford & Peters (2012)
Cone		Pinophyta; Cupressaceae	*Austrosequoia wintonensis*	Peters & Christophel (1978)
		Pinophyta; Araucarian	*Emwadea microcarpa*	Dettmann, Clifford & Peters (2009)
Leaf impression		Pterophyta	*Aff. Lygodium?*	Peters & Christophel (1978)
		Pterophyta; Osmundaceae	*Phyllopteroides macclymontae*	McLoughlin, Drinnan & Rozefelds (1995), McLoughlin, Pott & Elliott (2010)
		Pterophyta; ?Osmundaceae	*Cladophlebis sp.*	McLoughlin, Drinnan & Rozefelds (1995)
		Pterophyta	*Microphyllopteris sp.* cf. *M. gleichenoides*	McLoughlin, Drinnan & Rozefelds (1995)
		Pterophyta	*Sphenopteris sp.* cf*. S. warragulensis*	McLoughlin, Drinnan & Rozefelds (1995)
		Pterophyta	*Sphenopteris sp.*	McLoughlin, Drinnan & Rozefelds (1995)
		Pterophyta	*Indeterminate fern pinnule*	McLoughlin, Drinnan & Rozefelds (1995)
		Ginkgoales	*Ginko wintonensis*	McLoughlin, Drinnan & Rozefelds (1995)
		Pinophyta	*c.f. Austrosequoia wintonensis*	Peters & Christophel (1978); McLoughlin, Drinnan & Rozefelds (1995) McLoughlin, Pott & Elliott (2010)
		Pinophyta; Araucariaceae	*Araucaria sp.* *(scale-like leaves)*	Bose (1955) McLoughlin, Pott & Elliott (2010)
		Pinophyta; Araucariaceae	*Araucaria sp.* *(strap-like leaves)* *cf. A. mesozoica*	White (1974) Dettmann & Clifford (1992) McLoughlin, Drinnan & Rozefelds (1995)
		Pinophyta; Araucariaceae	*Araucaria cf. fletcheri* *(awl-like leaves)*	Bose (1955) McLoughlin, Pott & Elliott (2010)
		Pinophyta; ?Podocarpaceae	*Elatocladus plana*	McLoughlin, Drinnan & Rozefelds (1995)
		Pentoxylales	*Taeniopteris sp.*	McLoughlin, Drinnan & Rozefelds (1995)
		Bennetitales	*Otozamites sp. cf. Otozamites bengalensis*	McLoughlin, Pott & Elliott (2010)
		Bennetitales	*Ptilophyllum sp.*	McLoughlin, Pott & Elliott (2010)
		Magnoliophyta; Magnoliopsida	Angiosperm morphotype A	McLoughlin, Drinnan & Rozefelds (1995)
		Magnoliophyta; Magnoliopsida; Hamamelidae; ?Fagales	Angiosperm morphotype B	McLoughlin, Drinnan & Rozefelds (1995)
		Magnoliophyta; Magnoliopsida; Hamamelidae; ?Fagales	Angiosperm morphotype C ‘reminicient of’ *Nothofagus* from New Zealand (Pole, 1992)	McLoughlin, Drinnan & Rozefelds (1995)
		Magnoliophyta; Magnoliopsida; Hamamelidae; ?Fagales	Angiosperm morphotype D	McLoughlin, Drinnan & Rozefelds (1995)
		Magnoliophyta; Magnoliopsida; Hamamelidae; ?Fagales	Angiosperm morphotype E	McLoughlin, Drinnan & Rozefelds (1995)
		Magnoliophyta; Magnoliopsida; Hamamelidae; ?Fagales	Angiosperm morphotype F	McLoughlin, Drinnan & Rozefelds (1995)
		Magnoliophyta; Magnoliopsida; Hamamelidae; ?Fagales	Angiosperm morphotype G Similar to a NZ specimen tenatitvely assigned to Betulaceae (Pole, 1992)	McLoughlin, Drinnan & Rozefelds (1995)
		Magnoliophyta; Magnoliopsida; Hamamelidae; ?Fagales	Angiosperm morphotype H	McLoughlin, Drinnan & Rozefelds (1995)
		Magnoliophyta - dicotyledon	Angiosperm morphotype I	McLoughlin, Pott & Elliott (2010)
Other	Axes and nodal diaphrams	Equisitales	*Equisetites sp.*	Bose (1955), McLoughlin, Pott & Elliott (2010)
	False trunk	Pterophyta; Tempskyaceae	*Tempskya judithae*	Clifford & Dettmann (2005)
	Gammae	Hepaticales	*Marchantites marguerita*	Dettmann & Clifford (2000)

Taphonomy also must be accounted for, which may bias preservation of parts of a palaeo-landscape over others, and can result in a low apparent diversity of the death assemblage. The Turonian Raritan Formation (Christopher, 1979), for example, exhibits exquisite preservation in clays that lends itself to identification and quantification of the diversity of the flora in detail, while the Upper Cretaceous Winton Formation flora is variable in preservation, with many sites consisting of partial leaf impressions in silts or leaf impressions that are difficult to prepare whole (cf. QM L311). This argument cannot be made for all of the sites to which we have compared the Winton Formation flora herein; the plant materials in upper Albian Triton Point Member of Alexander Island, Antarctica, for example, exhibit similar preservation with no cuticle present and complete specimens rare (Cantrill & Nichols, 1996).

The effect of taphonomy can be investigated by comparing apparent diversity of different types of fossils. If we compare the angiosperm macrofossil record from the Upper Cretaceous portion of the Winton Formation to angiosperm pollen regionally from borehole studies, it seems pollen is recording a higher diversity (∼30 angiosperm palynomorphs; Burger, 1993). Yet difficulty assessing ages of the samples in light of new understanding of the temporal deposition of the Winton Formation, may mean this is a sum of 10 million years of deposition, artificially inflating the apparent diversity. Instead we compared different kinds of fossils at one site: pollen, wood and leaf impressions. For example, at QM L311, all of the wood is assigned to the podocarp, *Protophyllocladoxylon owensii* (Fletcher et al., 2014a) and in agreement, 87% of the pollen is referred to Podocarpaceae (Dettmann, Clifford & Peters, 2009). Pollen is considered to represent the diversity of the palaeo-landscape, even if not at a 1:1 ratio due to differences in pollen syndromes and low morphological distinctiveness within some groups (Birks et al., 2016). In a modern system, different genera of podocarp pollen were found to either truly represent the prevalence or under-represent the prevalence of podocarps in a system (Pocknall, 1978). Thus, although a taphonomic influence on the apparent diversity cannot be disproven, the congruence of microfossil and macrofossil evidence, and the fine sediments and preservation of microfossils at some localities, suggests other factors may also be causing the apparent low diversity.

Of the 12 factors that may influence diversity in modern systems compiled from Brown (1988), Roy et al. (1998), and Krebs (2001), two palaeoclimatic influences are proposed to have affected the diversity of the flora of the Upper Cretaceous portion of the Winton Formation in addition to taphonomy and sampling: super-annual climatic variability—in particular a potential multi-year cycle of wet and dry years—and related to this cycle, disturbance through flood events. Evidence for these influences in the environment comes from the sedimentary geology of the region (Tucker et al., 2013), growth indices in fossil wood (Fletcher, Moss & Salisbury, 2015), comparison of the fossil floral community composition with modern ecosystems (e.g., Holloway, 1954; Duncan, 1991; Duncan, 1993; Jaffré, 1995) and similarly low angiosperm diversity represented in the flood plain ecosystem of the coeval Mata Amarilla Formation (Iglesias et al., 2007). Whilst low diversity within a locality may frustrate some attempts to understand the palaeoenvironment e.g., diversity thresholds for using certain leaf physiognomic methods for palaeoclimatology (Spicer, Herman & Kennedy, 2005) the low diversity itself may be a palaeoenvironmental indicator, if one can account for the effects of sampling intensity and taphonomy. Future studies, particularly of well constrained palynological samples, may disprove the hypothesis posited here that the flora of the Upper Cretaceous portion of the Winton Formation was low diversity; however, evidence from the sediments, wood and analogous floral communities are consistent with a diversity depressed by disturbance in the form of floods, and super-annual climate variability.

## Fauna

**Figure 5:**
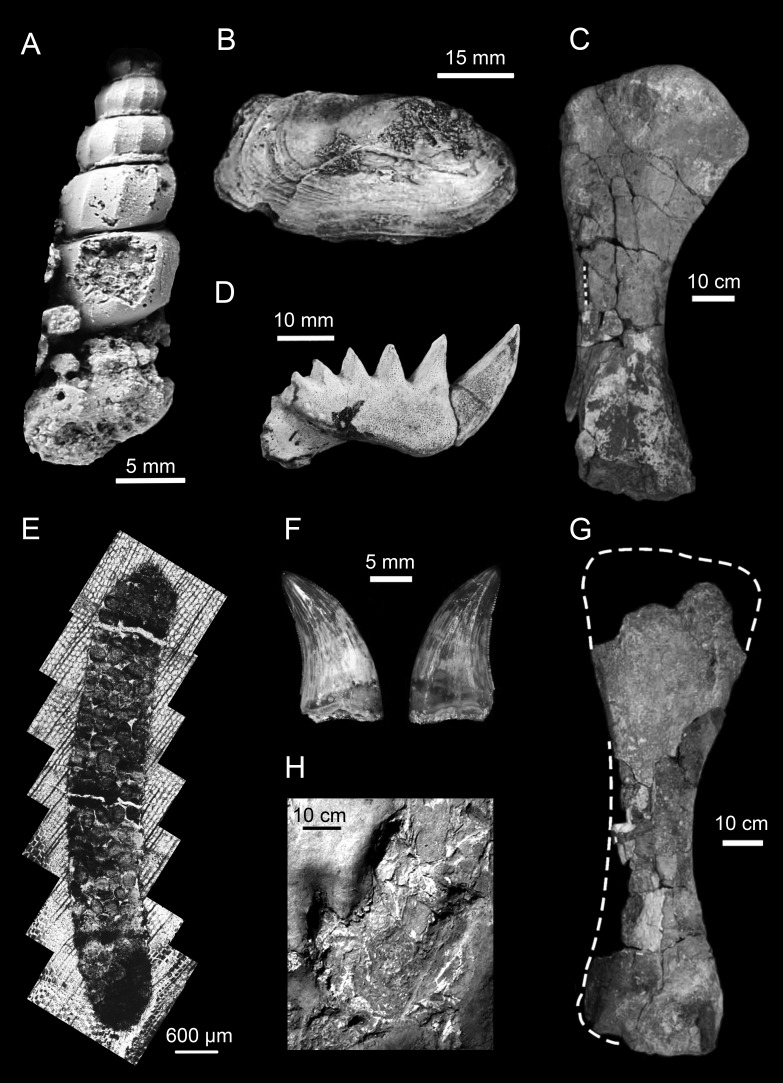
Representative animal fossils from the Upper Cretaceous portion of the Winton Formation. (A) *Melanoides sp.,* QM F49451 (Fig. 1; Cook, 2005); (B) *Alathyria jaqueti* Newton,** QM F103903 (Fig. 2M; Hocknull, 2000); (C) *Diamantinasaurus matildae* Hocknull et al., right humerus in lateral view, AODF 603 (Fig. 5D; Hocknull et al., 2009); (D) *Metaceratodus ellioti*, Kemp, QM F14971 (Photo: Rodney Berrell); (E) Probable oribatid mite trace, QM F44338 (Fig.2 ; Fletcher & Salisbury, 2014)^1^; (F) *Australovenator wintonensis* Hocknull et al. dentary teeth in labial view, AODF 604 (Fig. 20E, F; Hocknull et al., 2009); (G) *Wintonotitan wattsi* Hocknull et al., reconstructed right humerus in posterior view, QM F7292 (Fig. 16F; Hocknull et al., 2009); (H) cf. *Igunodontipus*, track 3 (L2), left pedal impression (contested; Fig. 5D; Romilio & Salisbury, 2014)^2^. ^1^Probable oribatid mite (Acari: Oribatida) tunnels and faecal pellets in silicified conifer wood from the Upper Cretaceous (Cenomanian–Turonian) portion of the Winton Formation, central-western Queensland, Australia, T.L. Fletcher and S.W. Salisbury, Alcheringa, copyright ©Association of Australasian Palaeontologists, reprinted by permission of Taylor & Francis Ltd, www.tandfonline.com on behalf of Association of Australasian Palaeontologists. ^2^Reprinted from Cretaceous Research, 51, A. Romilio and S.W. Salisbury, Large dinosaurian tracks from the Upper Cretaceous (Cenomanian–Turonian) portion of the Winton Formation, Lark Quarry, central-western Queensland, Australia: 3D photogrammetric analysis renders the ‘stampede trigger’ scenario unlikely, 186–207, Copyright (2014), with permission from Elsevier.

The current faunal list of the Upper Cretaceous (Cenomanian–Turonian) portion of the Winton Formation (Table 5; Fig. 5) includes evidence of invertebrates such as dragonflies and mecopteroids (Jell, 2004), freshwater bivalves (Hocknull, 1997; Hocknull, 2000), freshwater gastropods (Cook, 2005), and oribatid mites (Fletcher & Salisbury, 2014). Vertebrates are represented by turtles (Molnar, 1991), eusuchian crocodyliforms (Salisbury et al., 2006), semi-aquatic lizards (Scanlon & Hocknull, 2008), dipnoan lungfishes (Kemp, 1997), and dinosaurs such as small and large ornithopods (Thulborn & Wade, 1979; Thulborn & Wade, 1984)—represented by a single tooth (see Hocknull & Cook, 2008) and primarily through tracks (see Romilio & Salisbury, 2011; Romilio, Tucker & Salisbury, 2013; Thulborn, 2013; Romilio & Salisbury, 2014; Thulborn, 2017)—an indeterminate ankylosaurian (Leahey & Salisbury, 2013), titanosauriform sauropods (Coombs & Molnar, 1981; Molnar, 2001; Molnar & Salisbury, 2005; Hocknull et al., 2009; Molnar, 2010; Molnar, 2011; Poropat et al., 2015a; Poropat et al., 2015b; Poropat et al., 2016) and a megaraptoran theropod (Hocknull et al., 2009; Agnolin et al., 2010; Benson et al., 2010; White et al., 2012; White et al., 2013; White et al., 2016).

**Table 5:** Described elements of the fauna of the Upper Cretaceous portion of the Winton Formation.

Fossil type	Taxonomic group		Name	Reference
Body fossil	Mollusca	Bivalvia	*Alathyria jaqueti* *Megalovirgus wintonensis* *Hyridella macmichaeli*	Hocknull (1997) Hocknull (2000)
		Gastropoda	*Melanoides sp.*	Cook (2005)
	Insecta	Mecoptera	2 mecopteroid insects	Jell (2004)
		Odonata; Anisoptera	1 dragonfly	
	Osteichthyes	Sarcopterygii; Dipnoi	*Metaceratodus ellioti* *Metaceratodus wollastoni*	Kemp (1997)
	Reptilia	Squamata; Pythonomorpha; ?Dolichosauridae	cf. *Coniasaurus*	Scanlon & Hocknull (2008)
		Crocodiliformes; Mesoeucrocodylia; Eusuchia	Crocodyliformes indet.	Hocknull et al. (2009)
	Reptilia; Dinosauria	Megaraptora	*Australovenator wintonensis*	Hocknull et al. (2009)
		Titanosauriformes; Titanosauria	*Diamantinasaurus matildae*	Hocknull et al. (2009)Poropat et al. (2015b)
			*Savannasaurus elliottorum*	Poropat et al. (2016)
			unnamed	Coombs & Molnar (1981) Molnar (2001) Molnar (2011)
		Titanosauriformes; Somphospondyli	*Wintonotitan wattsi*	Coombs & Molnar (1981) Molnar (2001) Molnar & Salisbury (2005) Hocknull et al. (2009) Poropat et al. (2015a)
		Ankylosauria indet.	‘hypsilophodontid’	Leahey & Salisbury (2013)
		Ornithischia; Ornithopoda	[=Ornithopoda indet.]	Hocknull & Cook (2008)
Trace fossil	Insecta	Arachnid	Oribatid mite	Fletcher & Salisbury (2014)
	Dinosauria	Ornithischia; large-bodied ornithopod trace	cf. Iguanodontipus	Romilio & Salisbury (2011), Romilio & Salisbury (2014) Romilio, Tucker & Salisbury (2013) cf. Thulborn (2013) Thulborn & Wade (1979) Thulborn & Wade (1984) Thulborn (2017) White, Cook & Rumbold (2017)
		Ornithischia; small-medium-bodied ornithopod trace	*Wintonopus latomorum*	Thulborn & Wade (1984)
				Romilio, Tucker & Salisbury (2013)

As well as the climatic (see Palaeoclimatology) and hydrological (see Hydrology) needs of the animals for which we have modern analogues, the environmental preferences of some of the groups that have no modern analogue (e.g., non-avian dinosaurs) are also informative. Some nodosaurid ankylosaurs seem to have a broad range of ecological tolerances (Coombs & Deméré, 1996) and examples of ankylosaurians can be found living from polar latitudes (Molnar & Wiffen, 1994) to at least sub-tropical areas (Amiot et al., 2010) and from riparian (Wang et al., 1989; Amiot et al., 2010) to coastal environments (Molnar & Wiffen, 1994; Coombs & Deméré, 1996; Petti et al., 2010). However, what seems to link them is a preference for wet and well-vegetated environments (Vickaryous, Maryanska & Weishampel, 2004). Three titanosaurian and one non-titanosaurian titanosauriform are currently recognised from the Upper Cretaceous portion of the Winton Formation (Table 5). The presence of titanosaurians in the Upper Cretaceous portion of the Winton Formation is considered indicative of inland, fluvio-lacustrine environments, as opposed to coastal plains (Mannion & Upchurch, 2010). However, the presence of the non-titanosauriform somphospondyl *Wintonotitan wattsi* is contra this pattern. In their study of sauropod environmental preferences Mannion & Upchurch (2010) found eight localities that also had a mixed sauropod fauna. Although there is strong evidence of environmental preference, sauropod habitats were not strictly exclusive. Ornithopods have been shown to exploit a range of humid region habitats including wetlands (Nadon, 1993), lacustrine (Platt & Meyer, 1991), fluvial and riparian settings (Vila et al., 2013; Herne et al., 2018), and coastal environments (Diedrich, 2011) including lagoons (Vila et al., 2013). Both large and small ornithopods have been found in polar environments, with some potentially overwintering there, through to temperate climates (Rich & Rich, 1989; Chinsamy, Rich & Vickers-Rich, 1998; Chinsamy et al., 2012; Herne et al., 2018; Woodward, Rich & Vickers-Rich, 2018). Ornithopods appear to dominate high latitude to temperate localities, while saurichians, particularly sauropods, seem to dominate low latitude ‘tropical’ climate zones (Matsukawa, Lockley & Jianjun, 2006), although both extend beyond these ranges, as demonstrated by Antarctic (Cerda & Chinsamy, 2012) and Patagonian (Apesteguía, 2004; Salgado, Apesteguía & Heredia, 2005; Filippi & Garrido, 2012 and others) sauropods. Evidence from other localities suggests habitat partitioning between sauropods and ornithopods at the same latitude (Vila et al., 2013), but the co-occurrence of both is also known from the ichnological data (Salisbury et al., 2017). As such, ornithopods are not a strong indicator of palaeoenvironmental conditions.

There does not seem to be a clear pattern of distribution of megaraptoran theropods, but that they are almost always found in association with other dinosaurs (Smith et al., 2008; Novas et al., 2013), from polar localities (Molnar, Flannery & Rich, 1981; Benson et al., 2012) to lower latitudes (Bell et al., 2016). This suggests that as long as the environment can support the necessary sized prey animals, they will find it suitable and are unlikely a specific indicator of palaeoenvironment.

## Gaps and Future Directions

QM L311 comprises the most well-preserved flora of the Upper Cretaceous portion of the Winton Formation, and although investigation of this flora has been pursued (e.g., Peters & Christophel, 1978; Dettmann, Clifford & Peters, 2009; Dettmann, Clifford & Peters, 2012), undescribed material remains. Further investigation of its diversity and taphonomy may increase our understanding of the diversity of the Winton Formation and its place in the early evolution of the angiosperms. In addition, although much interest in the Winton Formation has centered around its faunal communities, prospecting for additional sites with similar lithology to QM L311 should continue in order to uncover similarly preserved, rich floral deposits.

The insect record is also very poor for the Winton Formation, likely due to the rapid destruction of specimens by weathering once exposed (Jell, 2004). The same kinds of exposures that could improve our floral record—fine matrix, newly exposed, silicified material—are also key sites to explore for insect body and trace fossils. Although some unreported specimens have been found, increased awareness among researchers and landholders that work in the region regarding the rarity and importance of these fossils may also improve the chances of increasing our understanding of this group given the current record comprises serendipitous finds uncovered during dam building (Jell, 2004).

Upper portions of the Winton Formation are the most likely to have been exposed to the extensive Cenozoic weathering that hampers understanding of the palaeoecology of this unit and surface exposures of the Winton Formation are very limited. Much of the detailed stratigraphic work to date, identifying Upper and Lower Cretaceous portions of the unit, has been conducted on data core logs or surface concretions. Many of the fossil-bearing localities, particularly those around Winton, do not have site-specific sedimentological and stratigraphic data associated with them, so broad scale stratigraphic correlations that include these sites are tentative and limited to a few key sites (namely Lark Quarry Conservation Park and Bladensburg National Park). This problem is exacerbated by extensive vertical faulting throughout the area. Continued investigation of the stratigraphy, sedimentology, and geomorphology of the region, along with the application of absolute dating methods to fossil-bearing localities within the Winton exposures, are key steps to guide future prospecting and to inform interpretation of the palaeoecology and palaeoenvironement of this important Australian Cretaceous formation.

## Conclusion: Palaeoenvironmental Reconstruction

When all of the current evidence—sedimentological, taphonomic, floristic, vertebrate, and invertebrate—is combined, a coherent yet complex image of the climate and environment of the Upper Cretaceous portion of the Winton Formation is apparent. The temperatures were warm, mesothermal, and probably similar to those of the sub-tropics today despite the palaeolatitude of ∼50° S. This was in keeping with globally high temperatures that supported forest in the southern polar regions during the Cretaceous (Cantrill & Falcon-Lang, 2001; Falcon-Lang, Cantrill & Nichols, 2001; Korasidis et al., 2016; Tosolini et al., 2018) at atmospheric CO_2_ as high as 1000 ppm (Franks et al., 2014). Precipitation was high and seasonal but not with extremes of wet and dry as is associated with the monsoon. The changing communities and ecology of the region over the 10 Ma deposition of the total Winton Formation was affected by the massive sediment loads derived from Whitsundays volcanism that infilled the inland sea (Tucker et al., 2016). This resulted in at least two distinct systems—an esturine, near-coastal system of higher energy, and a freshwater, inland, lower energy meandering river and flood plain—that must be considered separately.

The landscape that is preserved at the many sites where the Upper Cretaceous portion of the Winton Formation is exposed may be broadly continuous both temporally and stratigraphically, but in terms of their floral communities, they are heterogeneous. In some parts of the palaeolandscape, low diversity appears to be linked to a disturbance regime that may have comprised primarily flooding events. These flooding events were likely infrequent and possibly scouring in places when they occurred, leading to the establishment of long-lived podocarp stands that excluded other plants early in their establishment on the barren sediment. Other parts of the landscape were either less affected by flood events, or were affected at different times, such that their floral community was comprised of a different combination and abundance. These communities appear to have been more diverse, although not as diverse as would be expected of a forest climax community, and not as high or as diverse as at the other Late Cretaceous mid-latitude fossil floras to which it has been compared.

Comparing the range of communities recorded within the Upper Cretaceous portion of the Winton Formation to the other well-studied Australian Cretaceous plant localities, the older Albian Otway Basin flora, the Winton communities and their sediments are not as distinct as the six biofacies recorded in the Otway Basin (Tosolini et al., 2018). Whereas Tosolini et al. (2018) record broad leafed araucarians as part of a hinterland, high energy riverine and fire dominated systems, and flood plains, the outcroppings of the Upper Cretaceous portion of the Winton Formation represent a broadly continuous meandering river and flood plain system that dominated the landscape. The semi-aquatic reptiles, fish and invertebrates that were present are consistent with this system and accompanying minor lacustrine components. This created a landscape with high densities of vegetation necessary to support large, herd-living herbivorous dinosaurs in wet, riparian environments. In contrast to the small ornithopod and theropod polar dinosaurs that characterise the Otway fauna (Rich & Rich, 1989; Rich, Vickers-Rich & Gangloff, 2002), the Winton Formation was abundant in large sauropods, ornithopods and therapods perhaps reflecting differences in primary productivity not mirrored by diversity.

The disturbance events suggested to have shaped this palaeoenvironment may be linked to the influence of a ‘Pacific Decadal Oscillation’-like cycle affecting Queensland during the Late Cretaceous. This may have influenced both precipitation and temperature covariantly, as in modern climate systems. It is likely that precipitation varied between years, possibly on an oscillation of relative drought years and years of higher precipitation similar to the Pacific Decadal Oscillation. Thus, during the earliest part of the Late Cretaceous, when the rocks that now form the Upper Cretaceous portion of the Winton Formation were deposited, this part of eastern Gondwana was a dynamic and heterogeneous environment.

## Institutional Abbreviations

QM L: Queensland Museum Field locality
AOD L: Australian Age of Dinosaurs field locality

## Additional Information and Declarations

### Data Availability

The following information was supplied regarding data availability: This review article did not generate any data or code.
